# A Novel Functional Food Mix Attenuates Obesity Through Inhibition of Aldose Reductase, Advanced Glycation End-Products

**DOI:** 10.3390/foods15203635

**Published:** 2026-10-13

**Authors:** Krishna Kalyan Kalahasti, Pandarinath Savitikadi, S Sreenivasa Reddy, G Bhanuprakash Reddy

**Affiliations:** Biochemistry Division, ICMR-National Institute of Nutrition, Hyderabad 500007, India

**Keywords:** aldose reductase, advanced glycation endproducts, bioactives, functional food mix, inflammation, obesity, oxidative stress

## Abstract

Obesity is a multifactorial metabolic disorder associated with insulin resistance, oxidative stress, and excessive accumulation of advanced glycation end-products (AGEs). Emerging evidence suggests that aldose reductase (AR), a key enzyme of the polyol pathway, also contributes to obesity-associated metabolic dysfunction. In this study, we evaluated the potential of a functional food mix (FFM) containing *Emblica officinalis*, *Zingiber officinale*, *Curcuma longa*, *Piper nigrum*, *Cinnamomum zeylanicum,* and *Capsicum annuum* in specific proportions to prevent obesity by targeting AR inhibition and AGE accumulation. Male WNIN/GR-Ob obese rats were randomised into untreated obese, low-dose FFM (FF1), and high-dose FFM (FF2) groups, while lean littermates served as controls. Animals received the respective experimental diets for 6 months. Body weight, food intake, body composition (DEXA), biochemical parameters, carboxymethyl lysine (CML; a major AGE), and hepatic AR activity were assessed. FFM supplementation significantly attenuated body weight gain by 9 and 22% and reduced total fat mass by 8 and 23% with FF1 and FF2, respectively, with greater efficacy at the higher dose. Both doses lowered circulating leptin (*p* < 0.05), while FF2 significantly improved insulin sensitivity by reducing plasma insulin and HOMA-IR (*p* < 0.05). FFM also markedly decreased plasma CML concentrations and hepatic AR activity. Furthermore, treatment increased adiponectin levels, improved hypertriglyceridemia and hypercholesterolemia, and reduced lipid peroxidation (*p* < 0.05). Together, these findings suggest that the novel functional food mix exerts potent anti-obesity effects through inhibition of AR, suppression of AGE accumulation, and attenuation of oxidative stress.

## 1. Introduction

Obesity is one of the most prevalent public health issues among populations of all ages worldwide, contributing significantly to increased morbidity and mortality due to associated health complications. According to WHO 2022, more than one billion people are affected by obesity (BMI ≥ 30 kg/m^2^), including 890 million adults and 340 and 39 million adolescents and children, respectively [1]. The prevalence of generalised obesity (BMI ≥ 25 kg/m^2^) in the Indian subcontinent is 28.6%, affecting 242 million people [2]. It is a significant risk factor for non-communicable diseases like diabetes mellitus, cardiovascular diseases, hypertension, renal failure, cerebrovascular diseases, and vision disorders [3]. Obesity-related insulin resistance has pathophysiological features of low-grade inflammation, dysregulated adipokine production, oxidative stress, and accumulation of advanced glycation end-products (AGEs) [4]. In recent years, AGEs have emerged as important mediators of obesity and obesity-related metabolic disorders [5]. Clinical studies have revealed altered adipokine levels and increased circulating AGE-carboxymethyl lysine (AGE-CML) and its receptor, RAGE, in adipose tissue in obesity [6]. In addition to endogenous AGEs, dietary AGEs are linked to reduced insulin sensitivity and increased inflammation [7]. Recent research has highlighted the emerging role of aldose reductase (AR) in obesity and its associated complications through its effects on oxidative stress, inflammation, insulin resistance, endogenous fructose production, and adipose tissue dysfunction [8,9,10]. Furthermore, studies indicate that AR inhibition attenuates obesity, in which increased polyol pathway activity triggers hepatocyte reprogramming and promotes metabolic abnormalities [11]. Both genetic deletion and pharmacological AR inhibition have improved lipolysis and senescence markers in adipose tissue of high-fat diet-fed animals [12]. Both activation of the polyol pathway and accumulation of AGEs contribute to oxidative stress and inflammation.

With increasing prevalence, strategies to prevent and manage obesity and its related complications become critical. Besides lifestyle management and surgical interventions, the pharmacological treatment for obesity presents contradictions and side effects [13]. A safe and effective alternative to drugs for preventing obesity is plant-based spices and herbs traditionally used to treat many ailments. Inhibiting AR and AGEs is a potential preventive and therapeutic approach. Plant flavonoids and nutraceuticals modulate diverse cellular and physiological pathways by suppressing appetite, improving insulin sensitivity, and increasing thermogenesis. Clinical trials on various herbs and spices in people with obesity have shown reduced body weight, body mass index, waist-to-hip circumference, total body fat [14], and appetite [15]. However, the active constituent of an individual spice/herb may provide an insufficient effect and may require high dosages. Combining spices and herbs has been shown to have a more pronounced effect at sufficiently low concentrations [16]. Polyherbal formulations for obesity treatment have shown synergistic effects in improving insulin resistance and adipogenic factors [17] and in reducing body weight and steatosis in HFD animal models [18]. In our earlier studies, we screened and tested various functional foods that exhibited potent AR inhibition [19], anti-AGE activity [20], and antioxidant potential [21,22], which can prevent the progression of obesity and its related complications. Based on these studies, we formulated a functional food mix (FFM) with Amla, Turmeric, Black Pepper, Ginger, Cinnamon, and Capsicum in specific proportions to assess its anti-obesogenic effect in an obese rat model, with anticipated dose-dependent synergy.

## 2. Material and Methods

### 2.1. Functional Food Mix (FFM)

The constituents used to prepare FFM were high quality and purchased from local markets in Hyderabad, India. The pericarps of *Emblica officinalis* and *Zingiber officinale* rhizome were lyophilised and powdered, while dried *Curcuma longa* (rhizome), *Piper nigrum* (drupes), *Cinnamomum zeylanicum* (bark) and *Capsicum annuum* (fruits) were ground into powders. The anti-obesity functional food (FF) mix consists of powders of 1 g of *Emblica officinalis*, 0.25 g of *Curcuma longa*, 0.5 g of *Piper nigrum*, 1 g of *Cinnamomum zeylanicum*, 1.5 g of *Zingiber officinale*, and 0.5 g of *Capsicum annuum powder* (4.75 g) at a higher (FF2) dose and a 5-fold lower-dose of FF2 (0.95 g) constituted by powders of 0.2 g of *Emblica officinalis*, 0.05 g of *Curcuma longa*, 0.1 g of *Piper nigrum*, 0.2 g of *Cinnamomum zeylanicum*, 0.3 g of *Zingiber officinale*, and 0.1 g of *Capsicum annuum powder* as the FF1 dose; both (4.75 g or 0.95 g) were mixed into 100 g of rodent diet (AIN-93). The specific amounts of individual components in the mix were based on our earlier studies [23,24].

### 2.2. Animal Study

WNIN-GR/Ob is a spontaneously developed obese rat model from the Wistar rat colony at the ICMR-National Institute of Nutrition (ICMR-NIN) Animal Facility, which exhibits impaired glucose tolerance, hyperinsulinemia, hypertriglyceridemia, and leptin resistance [10,25,26]. We received 24 obese animals and their lean littermates, aged 2 months, from the colony and acclimatised them before grouping. Animal experiments were conducted in accordance with the guidelines of the Committee for Control and Supervision of Experiments on Animals (CCSEA). The Institutional Animal Ethics Committee of ICMR-NIN approved the animal experimentation protocols and procedures (IAEC Approval No: ICMR-NIN/IAEC/02/004-A/2020). The animals were housed under controlled conditions with a 12 h light/dark cycle, 50–60% humidity, and 22–25 °C room temperature, with free access to food and water. Lean animals (n = 6) served as lean controls, and obese animals (n = 18) were randomised by body weight into six animals per group in the Obese, Obese + FF1, and Obese + FF2 groups. Animals were fed the respective diet regimen for six months. We monitored food intake, body weight, and fasting blood glucose regularly until the end of the experiment. Body composition was analysed using dual-energy X-ray absorptiometry (DEXA; QDR Discovery A, S/N 82382, Hologic, Waltham, MA, USA) after 6 months. Blood was drawn from the retro-orbital plexus, and plasma was separated before the animals were sacrificed by CO_2_ asphyxiation. Liver tissue was dissected and snap-frozen in liquid nitrogen.

### 2.3. Biochemical Analysis

Animals were fasted overnight for 12 h, and fasting blood glucose was measured from tail-vein blood using a glucometer (ACCU-CHEK Active, Roche Diagnostics, Mannheim, Germany). Plasma triglycerides (Cat no: 700010029) and total cholesterol (Cat no: 700010013) were measured using colourimetric assay kits (Ecoline, Diasys Diagnostics India Pvt Ltd, Mumbai, India), following the manufacturer’s instructions. Plasma insulin levels were estimated using ELISA (Cat no: 10-1250-01, Mercodia AB, Uppasala, Sweden). HOMA-IR was calculated using the following formula to assess insulin resistance: [fasting insulin (µU/mL) × fasting glucose (mmol/L)]/22.5. Plasma carboxymethyl lysine (CML) (Ref: KLR1374, KRISHGEN Biosystems, Mumbai, India), leptin (890548, R&D Systems, Bio-Techne, Minneapolis, MN, USA), and adiponectin (Cat: DY3100-05, R&D Systems) were assessed using commercially available ELISA kits, following the manufacturer’s instructions. Lipid peroxidation in plasma was estimated as malondialdehyde spectrophotometrically using a thiobarbituric acid assay [27].

### 2.4. Aldose Reductase Enzyme Assay

Liver tissue was homogenised in phosphate buffer (20 mM, pH 7.4). The homogenate was centrifuged at 12,000× *g* for 20 min at 4 °C, and the supernatant was collected. Protein content in the soluble liver lysate was estimated using the modified Lowry method. Aldose reductase activity was measured using glyceraldehyde as the substrate, as described earlier [23], where the decrease in NADPH concentration was recorded spectrophotometrically at 340 nm on a UV–Vis spectrophotometer (Shimadzu UV 2600, Kyoto, Japan).

### 2.5. Statistical Analysis

The data presented were analysed using GraphPad Prism 9.0 and are represented as mean ± SD. One-way ANOVA with Tukey’s post hoc test was used to determine statistical significance among groups, and *p* < 0.05 was considered statistically significant.

## 3. Results

### 3.1. Effect of FFM on Food Intake and Body Weight

Food intake and body weights of the animals were monitored for 24 weeks. Average food intake of the untreated obese animal group compared to the lean control group was significantly higher (*p* < 0.01), and in FFM-treated obese animals, intake was decreased compared to that of untreated obese animals (Ob vs. FF1, *p* = 0.06; Ob vs. FF2, *p* = 0.07—Figure 1A). The untreated obese animals gained (nearly double) body weight compared to the lean control animals at the end of the experiment (*p* < 0.01), and supplementing the diet of obese animals with FFM significantly prevented weight gain by 9% in the FF1 group and 22% in the FF2 group compared to untreated obese animals, more so in the FF2 group (Ob vs. FF1, *p* = 0.3; Ob vs. FF2, *p* < 0.01—Figure 1B).

### 3.2. Effect of FFM on Body Composition

The body composition of the animals was determined using DEXA, as changes in body weight were observed at the end of the experiment due to FFM supplementation. Obese animals had significantly higher total mass (*p* < 0.01), surface area (*p* < 0.01), and fat mass (*p* < 0.01) than lean control animals. Obese animals supplemented with FF1 and FF2 showed a significant decrease in total fat mass of 8% and 23%, respectively, compared with untreated obese animals (FF2; *p* < 0.05, Figure 2).

### 3.3. Effect of FFM on Insulin and Leptin Resistance

Fasting glucose was measured regularly, and there were no significant changes in fasting glucose levels between groups (Figure 3A). Serum leptin, insulin and HOMA-IR were estimated at the end of the experiment. As previously reported, untreated obese animals showed significantly higher insulin (*p* < 0.01) and leptin (*p* < 0.01) levels than lean controls. These findings indicate that this animal model exhibits insulin and leptin resistance, as well as impaired glucose tolerance (Figure 3B,C). Supplementation of FFM to obese rats decreased serum insulin and HOMA-IR by 74% and 61%, respectively; the effect was profound with FF2 (*p* < 0.05) compared to FF1 (*p* = 0.06), and leptin levels were reduced by 35.9% and 39.2% with both doses of FFM (*p* < 0.05), indicating a preventive effect on insulin and leptin resistance (Figure 3).

### 3.4. Effect of FFM on Accumulation of AGE and Aldose Reductase Activity

Carboxymethyl lysine (CML), a predominant AGE, contributes to insulin resistance and adipocyte dysfunction. Serum levels of CML were significantly higher in untreated obese animals compared to lean control animals (*p* < 0.01). FFM treatment of obese animals decreased CML by 25% and 18% with FF1 and FF2, respectively, compared with untreated obese animals (*p* < 0.01, Figure 4A). Increased AR activity in the liver is a driver of de novo lipogenesis. Aldose reductase activity in liver tissue was reduced in the FFM-treated animals by 24.3% and 31.7% with FF1 and FF2, respectively, compared with the untreated obese animals, and the effect was statistically significant in the FF2 group (*p* < 0.05, Figure 4B).

### 3.5. Effect of FFM on Adiponectin Levels, Lipid Profile, and Malondialdehyde

Since there was improved insulin resistance in the obese animals treated with FFM, we estimated serum adiponectin levels. Adiponectin levels of untreated obese animals were significantly lower compared with lean control animals (*p* < 0.05), and FFM supplementation at both doses improved adiponectin levels by 148% and 306% in obese rats with FF1 and FF2 doses, while a statistically significant change was observed with FF2 (*p* < 0.05, Figure 5A). Serum triglyceride and total cholesterol levels were higher in untreated obese animals compared to lean control rats (*p* < 0.01), indicating hyperlipidaemia, and FFM supplementation in obese animals significantly decreased total triglyceride levels by 36% and 47.6% and cholesterol levels by 37.7% and 33%, respectively, with FF1 and FF2 (*p* < 0.05, Figure 5B,C). Levels of malondialdehyde (MDA), a lipid peroxidation product, were determined in plasma. Untreated obese animals had higher MDA levels than lean controls (*p* < 0.05), and the FF2 treatment significantly decreased MDA by 23.8% in obese animals (*p* < 0.05, Figure 5D).

## 4. Discussion

The prevalence of obesity and the associated disease burden is rapidly increasing worldwide. For a multifactorial disease like obesity, intervention is challenging. The complex pathogenesis of obesity indicates the need for different intervention strategies to confront this problem. Common strategies for the prevention and management of obesity include lifestyle modification, dietary restriction, pharmacotherapy, and bariatric surgery. Obesity treatment guidelines suggest that the intervention should not only address weight loss but also adiposity-associated complications [28]. Dietary intervention with physical exercise has been shown to be more effective than diet or exercise independently [29,30]. Medications approved for obesity that are given as adjuncts to dietary interventions have been shown to be very effective in weight loss, hypertension, and glucose-lowering but have a higher risk of gastrointestinal and psychological problems [31,32]. Recently, functional foods have been used to manage obesity because they contain a wide variety of bioactive phytochemicals that affect biochemical and metabolic pathways linked to obesity. Phytoconstituents exert anti-obesity effects by acting through various mechanisms, including inhibition of adipocyte differentiation and adipogenesis, inhibition of pancreatic enzymes, and regulation of leptin and ghrelin [33,34].

In this study, we formulated a novel polyherbal mix with Amla, Black Pepper, Cinnamon, Ginger, Turmeric and Capsicum and investigated its effects on obesity. Some of the individual constituents of this mix were reported for their role in anti-obesity in previous studies [17,18,35], and we have also reported them for their potential in preventing diabetic complications in animal models [21]. We reported that Amla extract and its bioactive β-glucogallin inhibit sorbitol accumulation and AR activity in a rat model [36]. Ellagic acid and procynadin B2, present in Cinnamon, exerted potent anti-glycation properties and inhibited accumulation of AGEs [37,38]. Capsaicinoids have been shown to increase fat oxidation and reduce abdominal adiposity [35]. Hence, we developed this formulation to achieve synergistic efficacy and explored its effect on AR inhibition and AGE accumulation, as well as, in turn, preventing obesity. Another version of this formulation has shown AR inhibition, anti-glycation potential, and the antioxidant properties of bioactives present in FFM in the context of diabetes-associated complications [23,24]. Feeding FFM to the obese rats prevented weight gain, with no significant change observed in food intake. However, DEXA results revealed lower free fat mass in the treated obese animals. Recent studies have shown the role of AR in metabolic abnormalities such as de novo lipogenesis and insulin resistance and that inhibition of AR attenuates hepatocyte reprogramming [11]. In obesity, mTOR promotes lipid biosynthesis by upregulating the transcription factor SREBP [39]. Insulin resistance promotes fatty acid synthesis and reduces glycogen formation in the liver. Impaired insulin and leptin levels lead to increased food intake and weight gain and are often seen in obesity and type 2 diabetes. We found that FFM treatment lowered plasma leptin and insulin levels in obese animals and decreased HOMA-IR, indicating improved insulin resistance. AR inhibition may reduce metabolic flux through pathways promoting endogenous fructose production and hepatic lipogenesis. Phytochemicals from natural food sources have been proven to inhibit mTOR/SREBP and adipogenesis and improve glucose dysregulation and thermogenesis in diet-induced obesity models [21,40,41,42].

Increased CML and malondialdehyde (MDA), markers of AGE and lipid peroxidation, respectively, observed in the plasma of obese individuals have been reported to cause oxidative stress and inflammation and contribute to insulin resistance [6,43]. The simultaneous reduction in CML and MDA suggests that FFM may interrupt the reciprocal interaction between glycoxidative stress and metabolic dysfunction. AGE accumulation can promote oxidative stress and inflammatory signalling, while oxidative stress can further accelerate glycation and AGE formation. Therefore, the observed reductions in CML and MDA may represent complementary, rather than independent, effects of the formulation. Clinical studies that are focused on dietary AGEs also reported that lower consumption of AGEs in the diet improved body weight and insulin sensitivity (HOMA-IR) in individuals with obesity [7,44]. Higher concentrations of plasma-soluble RAGE (sRAGE) were observed in normal compared to obese individuals, where increased sRAGE concentration attenuates AGE-RAGE signalling. Contrarily, calorie restriction in individuals with morbid obesity has improved insulin sensitivity, inflammation and dyslipidemia; however, plasma sRAGE levels were not improved [45]. Adiponectin is a biomarker for obesity and has a protective role as an insulin sensitiser [46]. Adiponectin levels are reported to be lower in individuals with obesity due to insulin resistance [47], adipogenesis, inflammation, and AGE/RAGE activation [48]. FFM treatment in obese animals improved plasma adiponectin levels and attenuated CML and MDA levels. The increase in adiponectin is particularly relevant because adiponectin has insulin-sensitising, anti-inflammatory, and lipid-regulatory properties. The concurrent increase in adiponectin with reductions in insulin resistance, triglycerides, cholesterol, CML, and MDA suggests a coordinated improvement in adipose tissue metabolic function. This may indicate that FFM not only reduces fat accumulation but also improves the functional quality of adipose tissue.

## 5. Conclusions

In the present study, we report that a unique functional food mix exerts a coordinated metabolic effect beyond simple suppression of body weight gain by inhibiting AR and AGE accumulation, with antioxidant and anti-inflammatory effects as the targeted mechanisms. These findings also support the possibility that modulating the AR–AGE–oxidative stress axis contributes to improved obesity-associated metabolic dysfunction. The increased efficacy achieved through the synergy of the compounds in the mix and their action across multiple mechanisms makes FFM a promising natural therapeutic approach, implemented alongside other strategies in the management of obesity and its metabolic complications. Further mechanistic and clinical studies are warranted to validate its translational potential.

## Figures and Tables

**Figure 1:**
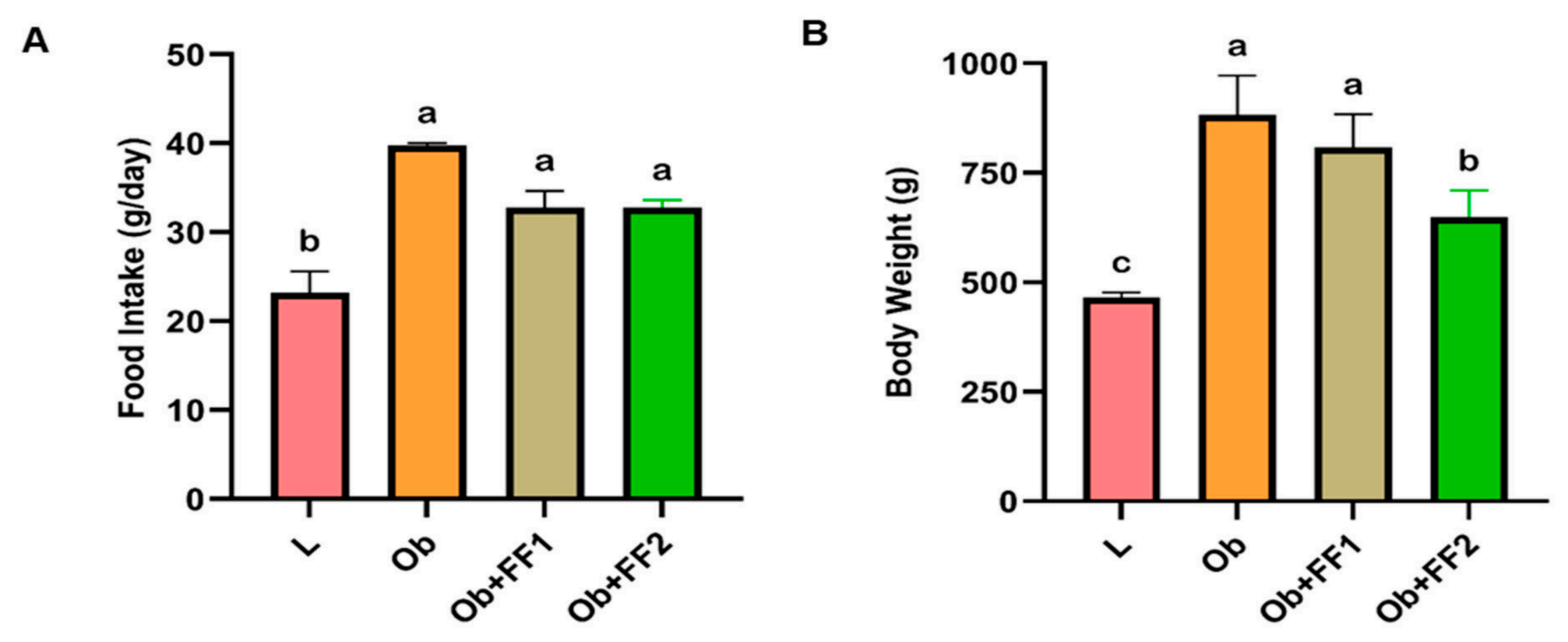
Food intake and body weight of animals in the experimental groups. (**A**) Average food intake and (**B**) average body weight at the end of the experiment. Different superscripts on the bars indicate a significant difference between the treated and untreated groups using one-way ANOVA with Tukey’s post hoc test. Data are represented as mean ± SD, n = 6, *p* < 0.05.

**Figure 2:**
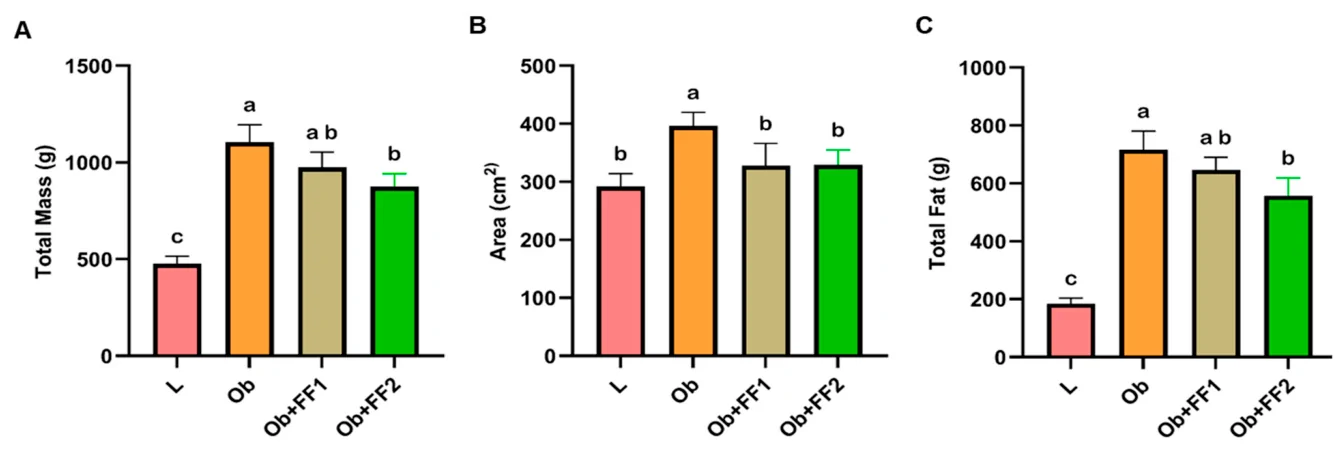
Effects of FFM on body composition of the animals. (**A**) Total mass, (**B**) surface area, and (**C**) total fat mass. Different superscripts on the bars indicate a significant difference between the treated and untreated groups using one-way ANOVA with Tukey’s post hoc test. Data are represented as mean ± SD, n = 6 per group, *p* < 0.05.

**Figure 3:**
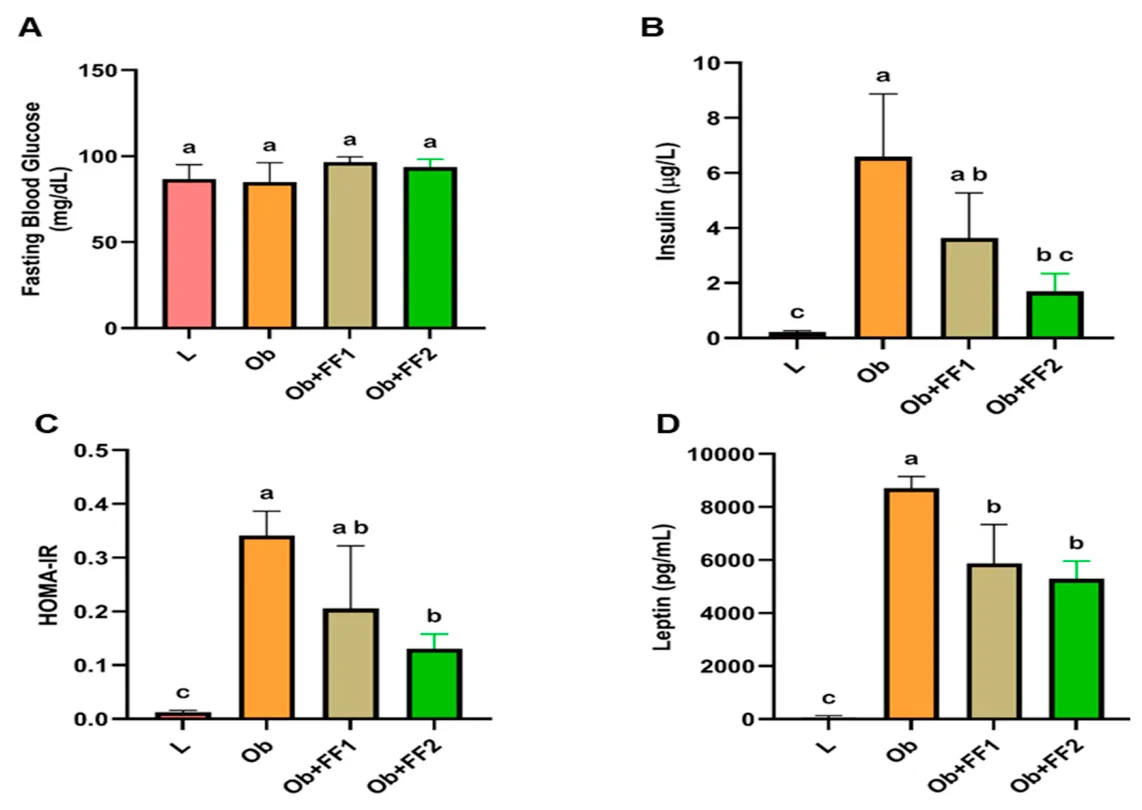
FFM improved insulin and leptin resistance. Plasma levels of blood glucose (**A**), insulin (**B**), HOMA-IR (**C**), and leptin levels (**D**). Different superscripts on the bars indicate a significant difference between the treated and untreated groups using one-way ANOVA with Tukey’s post hoc test. Data are represented as mean ± SD, n = 6 per group, *p* < 0.05.

**Figure 4:**
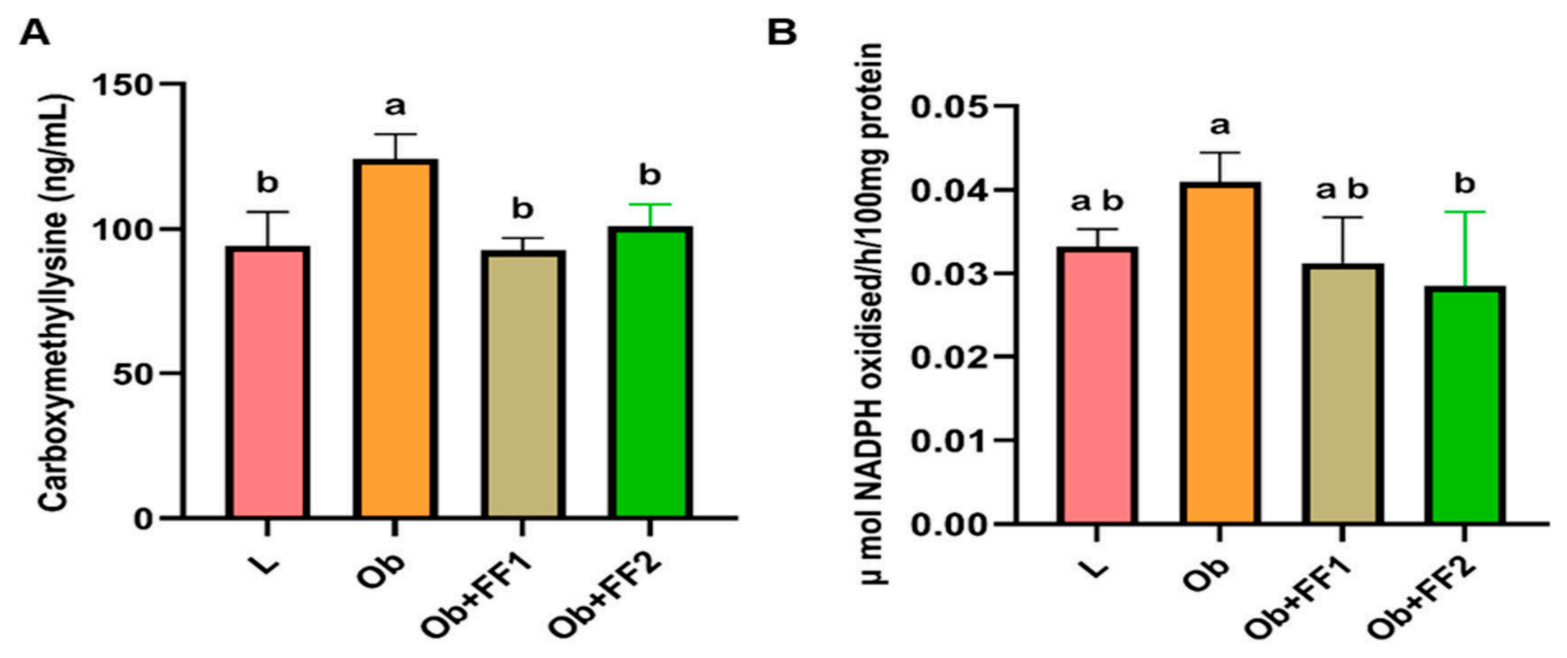
Effects of FFM on AGEs and aldose reductase activity. Plasma levels of carboxymethyl lysine (**A**) and aldose reductase activity in liver (**B**). Different superscripts on the bars indicate a significant difference between the treated and untreated groups using one-way ANOVA with Tukey’s post hoc test. Data are represented as mean ± SD, n = 6 per group, *p* < 0.05.

**Figure 5:**
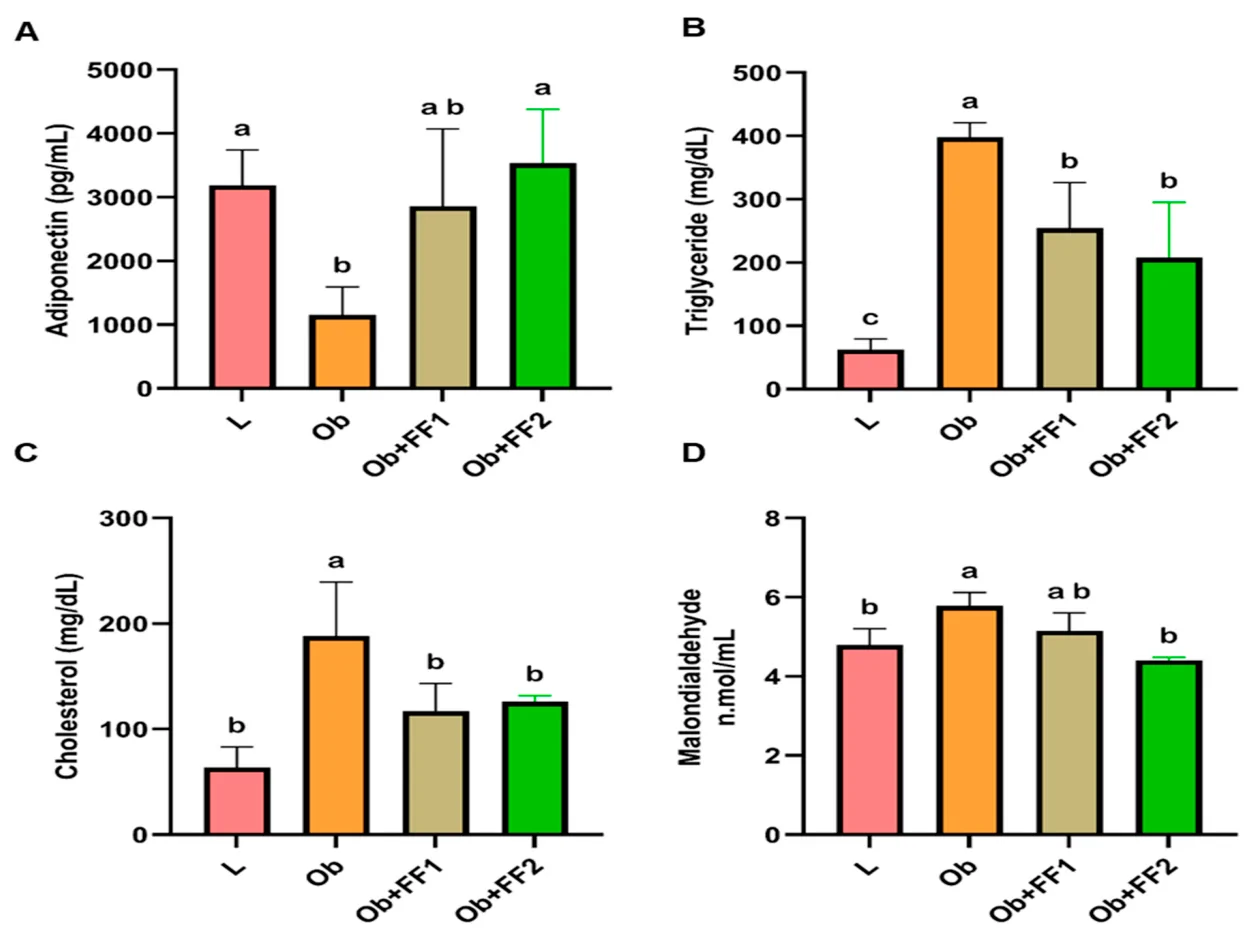
Effects of FFM on adiponectin, lipid profile and malondialdehyde. Plasma levels of adiponectin (**A**), triglycerides (**B**), cholesterol (**C**), and malondialdehyde (**D**). Different superscripts on the bars indicate a significant difference between the treated and untreated groups using one-way ANOVA with Tukey’s post hoc test. Data are represented as mean ± SD, n = 6 per group, *p* < 0.05.

## Data Availability

The original contributions presented in the study are included in the article, further inquiries can be directed to the corresponding author.
